# Bullying Among Tunisian Middle School Students: the Prevalence, Psychosocial Associated Factors and Perceived Involvement of Parents, Teachers and Classmates

**Published:** 2018-05-05

**Authors:** Jihene Sahli, Manel Mellouli, Meriam El Ghardallou, Manel Limam, Mouna Gallas, Asma Ammar, Ali Mtiraoui, Thouraya Nebli Ajmi, Chekib Zedini

**Affiliations:** ^1^Department of Familial and Community Medicine; Faculty of Medicine of Sousse; Research Laboratory “LR12ES03”. University of Sousse, Tunisia

**Keywords:** Bullying, Psychosocial factors, Schools, Students

## Abstract

**Background:** Bullying is a serious public health concern remarkably common among youth. Involvement in bullying can lead to deleterious effect on the emotional well-being of pupils. The aim of this study was to assess the prevalence of bullying, its psychosocial associated factors and the perceived involvement of parents, teachers, and classmates to counteract this behavior.

**Study design:** A cross-sectional study.

**Methods:** We conducted this study in 2015 among a representative multistage sample of 1584 students enrolled in middle schools in the Region of Sousse using the revised Olweus Bully/Victim Questionnaire. It assesses the prevalence of bullying and covers qualitative details of bullying including psychosocial factors and perceived efforts of others to counteract bullying.

**Results:** 11.7% of respondents were classified as pure victims, 7.8% as pure bullies, 3.2% as bully-victims and 75.5% as bystanders. Compared to other groups, the bully-victims were less likely to report a feeling of empathy and liking school. They were more likely to be afraid of being bullied, aggressive and to have fewer friends in the class. Only 30.3% of the victims indicated that they told someone about being bullied. The majority of the middle school students perceived that classmates (54.1%) and teachers (39.5%) did nothing to counteract bullying.

**Conclusions:** Information about bullying is critical and must be gathered before effective intervention is planned. Parents, teachers and students should learn effective ways to handle the bullying problem since the most effective programs are comprehensive targeting students, schools, families and the community.

## Introduction

Bullying is a subcategory of interpersonal aggression defined by three central features: 1) an intention to inflict harm on another person; 2) a repetition of the negative behavior over time, and 3) a physical or psychological imbalance in strength between perpetrators and victims ^1^.

Bullying can take many forms: physical (e.g., attacking, hitting, and biting), verbal (e.g., name-calling), relational (e.g., gossiping, social exclusion), and cyberbullying (Through electronic means). Physical and verbal bullying are often considered as direct bullying and relational bullying to be indirect bullying^1^. Bullying is often considered as a relationship between individuals taking multiple roles (The bully, the victim, the bully/victim and bystander)^2^.

Although still viewed as an inevitable part of growing up, bullying is a serious public health concern remarkably common among school children^3,4^. Bullying has generally been shown to be most prevalent in middle schools ^5^. The transition from elementary to middle school is an important developmental task for early adolescents ^6^. It is a critical period where youth are negotiating new peer groups and use bullying as a means to achieve social dominance ^7^.

Bullying can lead to numerous adverse outcomes including a lower level of school bonding and liking, a feeling of fear and being unsafe in school, a higher rate of school refusal or absenteeism, poor school achievement, subsequent violent behavior and a variety of mental disorders comprising attempted and completed suicide ^6,8^.

These dangerous impacts on youth well-being add greater urgency to the search for appropriate strategies and approaches to prevent bullying among schoolchildren. The research literature on bullying prevention has clearly indicated that focusing only on the behavior to be eliminated is less effective than having a simultaneous focus on constructing a positive context taking into account the child, the family, the school and the community ^9^. In fact, increasing the level of empathy and the involvement of parents, teachers, and classmates, when bullying occurs, are among the effective ways to diminish and stop this behavior ^10,11^.

In contrast with developed countries, little research has been conducted on bullying in developing countries such as Tunisia. This study was conducted in Tunisia among Tunisian middle school students aim to give more insight into the phenomenon of bullying, its psychosocial associated factors and the perceived involvement of parents, teachers, and classmates to counteract this behavior.

## Methods

This is a cross-sectional study conducted in 2015 among a representative sample of students enrolled in colleges in the governorate of Sousse. The target population of this study was restricted to students in the 7^th^, 8^th^, and 9^th^, with modal ages of 11 to 15 yr, enrolled in middle schools in the Region of Sousse during the school year of 2015/2016. We calculated the sample size according to the following formula: n = Zα_/2_² × (*P* × q) / i² = 994 (n = minimum sample size, Zα_/2_= 1.96 (α = 0.05), *P* = 0.369 ^12^; q = 0.631, i = 0.03). Informed consent was taken from the participants before the study.

To select randomly our population, we used a three-stage probability sampling technique. In the first stage, primary sampling units were the 16 delegations of the Region of Sousse, from which we selected eight delegations. In the second step, we randomly selected 14 schools with a probability proportional to the number of colleges in each delegation. For reasons of feasibility, in the third step, we randomly selected one from five classes having courses the day of the survey. All students in selected classes who were present the day of the survey and accepted to respond to the questionnaire were included. Parental or student refusal to participate was a non-inclusion criterion to the current study.

We used the revised Olweus Bully/Victim Questionnaire, which is a 39 item self-administered questionnaire. A good reliability and validity is reported already ^13^. Two questions “item 4” and, “item 24,” asked respectively how often participants had either been bullied (victimization) or bullied others (bullying) in the past couple of months. These two global questions are each followed by nine more specific questions about how often particular forms of bullying and victimization have occurred. Response options were “none”, “only once or twice”, “2 or 3 times a month”, “about once a week”, and “several times a week” ^13^.

“2 or 3 times per month” was considered the cutoff point for classifying a student as a victim/non-victim or a bully/non-bully for the global and the specific questions^13^. Then, we classified the students into four groups: (1) those who were both victims and bullies (bully-victims), (2) those who were victims only (pure victims), (3) those who were bullies only (pure bullies), and (4) those who were neither victims nor bullies (not involved or bystanders).

The questionnaire covers also qualitative details of bullying including the psychosocial factors, and the perceived level of the involvement of parents, teachers, and classmates to counteract bullying when it occurs. Pupils were also asked if they told someone about their being bullied or if someone talked with them about their bullying others. Psychosocial factors included school liking, fear of being aggressed, tendency to aggressiveness, having friends in the class and classmates’ empathy. In the questionnaire, these factors were assessed by Likert scale questions and then categorized into dichotomous variables. The involvement of teachers, parents, and classmates were assessed by questions assessing how much they did to counteract bullying or help the bullied.

Categorical variables were presented by their absolute and relative valid frequencies. A two-tailed *P* -value of <0.05 was considered the threshold for statistical significance. Chi-square test was used to compare frequencies. Statistical analysis was performed using the SPSS 21.0 software (Chicago, IL, USA)

## Results

We enrolled 1584 middle school students. Among the respondents, 51.3% (n = 807) were boys and 48.7% (n=765) were girls (12 did not respond to the question relative to gender); and 43.8% (n = 688) were in the 7^th^ grade; 28.7% (n = 451) in the 8^th^ grade and 27.5% (n = 433) in the 9^th^ grade. Their age ranged from 11 to 15 yr old.

The prevalence of bullying and victimization was 16.0% (n=248) [95% CI: 14.2, 17.8] and 11.3% (n=170) [95% CI: 9.7, 12.9] respectively. From these global measures of bullying and victimization, four groups were identified, 11.7% (n=179) were classified as pure victims, 7.8% (n=118) were classified as pure bullies, 3.2% (n=50) were classified as bully-victims and 75.5% (n=1135) were classified as bystanders (Table 1).

**Table 1:** Prevalence of bullying/victimization and different roles of the middle school students by gender, Sousse (Tunisia), 2015

**Variables**	**Boys, n (%)**	**Girls, n (%)**	***P*** **value**
Prevalence of bullying and victimization		
Victimization	132 (16.7)	112 (14.9)	0.340
Bullying	98 (12.9)	72 (9.8)	0.063
Different roles in the bullying behavior		
Pure victim	97 (12.4)	79 (10.6)	0.283
Bully-victim	25 (3.2)	25 (3.3)	0.874
Pure bully	71 (9.2)	47 (6.3)	0.036
By-stander	560 (73.2)	572 (78.2)	0.023

Being called mean names, made fun of or teased in a hurtful way (55.6%) was the most prevalent specific form of being bullied. There was no significant difference between boys and girls in the specific ways of being bullied. Bullying with mean names or comments about race or color was the most prevalent specific way of bullying others (21.7%). Compared to boys, girls were more likely to bully others by calling mean names, making fun of or teasing in a hurtful way and by threatening or forcing others to do things they did not want to do (Table 2).

**Table 2:** Different specific ways of bullying and victimization according to the bullies and the victims, Sousse (Tunisia), 2015

**Items**	**Boys, n (%)**	**Girls, n (%)**	***P *** **value**	**Total**
**Specific ways of being bullied (according to the victims)**				
“I was called mean names, was made fun of, or teased in a hurtful way”	79 (59.8)	56 (50.5)	0.142	135 (55.6)
“Other students let me out of things on purpose, exclude me from their group of friends, or completely ignored me”	43 (33.1)	32 (29.4)	0.537	75 (31.4)
“I was hit, kicked, pushed, shoved around, or locked indoors”	47 (35.9)	35 (31.8)	0.508	82 (34.0)
“Other students told lies or spread false rumors about me and tried to make others dislike me”	52 (40.0)	40 (36.4)	0.564	92 (38.3)
“I had money or other things taken away from me or damaged”	44 (33.6)	32 (29.1)	0.454	76 (31.5)
“I was threatened or forced to do things I didn’t want to do”	31 (23.7)	17 (16.2)	0.156	48 (20.3)
“I was bullied with mean names, comments about my race or color”	44 (33.8)	33 (30.8)	0.623	77 (32.5)
“I was bullied with mean names, comments, or gestures with a sexual meaning”	41 (31.5)	34 (31.8)	0.969	76 (31.4)
**Specific ways of bullying others (according to the bullies)**				
“I called another student mean names, made fun of or teased him or her in a hurtful way”	8 (11.0)	13 (24.1)	0.049	21 (16.5)
“I kept him or her out of things on purpose, excluded him or her from my group of friends or completely ignored him or her”	5 (6.2)	7 (12.1)	0.222	12 (8.6)
“I hit, kicked, pushed and shoved him or her around or locked him or her indoors”	8 (11.8)	9 (16.1)	0.488	17 (13.7)
“I spread false rumors about him or her and tried to make others dislike him or her”	2 (2.4)	5 (8.8)	0.119	7 (5.0)
“I took money or other things from him or her or damaged his or her belonging”	4 (4.8)	4 (6.9)	0.716	8 (5.6)
“I threatened or forced him or her to do things he or she didn’t want to do”	11 (11.8)	19 (27.5)	0.011	30 (18.5)
“I bullied him or her with mean names or comments about his or race or color”	17 (18.5)	18 (26.1)	0.247	35 (21.7)
“I bullied him or her with mean names, comments, or gestures with a sexual meaning”	16 (17.2)	7 (10.4)	0.229	23 (14.4)
“I bullied him or her with mean or hurtful messages, calls or pictures, or in other ways on my mobile phone or over the Internet (computer)”	12 (14.0)	15 (22.1)	0.189	27 (17.5)

Compared to other groups, the bully-victims were less likely to report a feeling of empathy (38.3%, *P* <10^-3^) and liking school (30.0%, *P* <10^-3^). They were more likely to be afraid of being bullied (49.0%, *P* <10^-3^), aggressive (56.2%, *P* <10^-3^) and to have only one friend (30.6%, *P* =0.002) (Table 3).

**Table 3:** Psychosocial factors and different participant role in the bullying behavior, Sousse (Tunisia), 2015

	**Pure** **victims** **n (%)**	**Pure** **bullies** **n (%)**	**Bully-** **victim** **n (%)**	**Bystanders** **n (%)**	***P*** **value**
Empathy					0.001
Yes	138 (78.4)	78 (69.0)	29 (61.7)	896 (81.5)	
No	38 (21.6)	35 (31.0)	18 (38.3)	204 (18.5)	
Liking school					0.001
Yes	143 (82.2)	96 (82.1)	35 (70.0)	1042 (93.0)	
No	31 (17.8)	21 (17.9)	15 (30.0)	7 (7.0)	
Tendency to aggressiveness					0.001
Yes	42 (25.5)	63 (55.8)	27 (56.2)	182 (16.7)	
No	123 (74.5)	50 (44.2)	21 (43.8)	907 (83.3)	
Fear to be bullied					0.001
Yes	88 (54.3)	79 (69.9)	24 (49.0)	780 (71.8)	
No	74 (45.7)	34 (30.1)	25 (51.0)	307 (28.2)	
Having more than one friend					0.002
Yes	49 (27.2)	20 (17.1)	15 (30.6)	198 (17.5)	
No	128 (72.3)	97 (82.9)	34 (69.4)	932 (82.5)	

Among the respondent victims (n=235), 30.3% (n=109) indicated that they told someone about being bullied, it was a friend in 67.9% of cases. Only 11.7% and 20.9% of the bullies have reported that they talked several times about their bullying others with teachers and parents/guardians, respectively (Table 4).

**Table 4:** Talking about bullying and victimization with someone according to the bullies and the victims

**Variables**	**Number**	**Percentage**
**Talking about victimization (n=235)**	109	30.3
Person being told of victimization (n=109)		
The class teacher	30	27.5
Another adult at school	43	39.4
Parents/guardians	45	41.3
Brother(s) or sister(s)	25	22.9
Friend(s)	74	67.9
Somebody else	26	23.8
**Talking about bullying others (n=163)**		
With class teacher or another teacher		
Only once	25	15.3
Several times	19	11.7
Parents/guardians		
Only once	32	19.6
Several times	34	20.9

The students perceived that classmates and teachers did nothing to counteract the bullying behavior in 54.1% and 39.5% of cases respectively (Table 5).

**Table 5:** The perceived involvement of teachers, parents and classmates to counteract the bullying, Sousse (Tunisia), 2015

**Variables**	**Number**	**Percentage**
Trying to stop bullying by teachers (n=1451)^a^		
Almost never	565	39.5
Once a while	217	15.2
Sometimes	209	14.6
Often	202	14.1
Almost always	238	16.6
Trying to stop bullying by students (n=1483)^a^		
Almost never	803	54.1
Once a while	277	18.7
Sometimes	242	16.3
Often	98	6.6
Almost always	63	4.2
Contacting school to stop bullying by parents (n=144)^b^	
No	95	66.0
Once	33	22.9
Several times	16	11.1
Efforts done by teachers to counteract bullying (n=1431)^b^	
Little or nothing	665	45.8
Fairly little	300	20.7
Somewhat	230	15.9
A good deal	143	9.9
Much	113	7.8

^a^ According to all participants ^b^ According to the victims

## Discussion

We aimed to highlight the extent and the nature of the bullying behavior among middle school students in the Tunisian context and to identify its associated psychosocial factors. Our study highlighted also the perceived level of involvement of teachers, parents, and classmates to handle this behavior in school setting. The prevalence of victimization and bullying was 16.0 % and 11.3%, respectively without a significant difference between boys and girls.

The prevalence of bullying and victimization varied greatly across countries from 5% to greater than 80% ^14^. Overall, 10% to 33% of children were reported being bullied^15^. Disparate assessment approaches, as well as differences across individuals, contexts, and cultures, may account for the variation in the school bullying prevalence ^15^.

Based on our results, there was no gender difference in bullying and victimization, however, researchers have indicated that typically boys report more bullying than girls, and girls report more victimization ^15,16^. Bullies and victims were not necessarily distinct groups of individuals. In addition to pure bullies and pure victims, there is a distinct group of adolescents who victimized and bully others and labeled as bully-victims ^17^. The bystander group is represented by students who reported not bullied by others either bullied others. The bully-victim group was the less prevalent group in the current work (3.2%). The prevalence of bully-victims was varied from 0.4% to 29% in studies using self-reports^18^. Consistent with previous research^17,19,20^, boys were more likely to be pure bullies.

In relation to the specific ways of bullying, verbal bullying with mean names or comments about race or color was the most prevalent specific way of bullying others according to the bullies. The gender analysis has shown that girls were more likely to bully others verbally by calling mean names, making fun of or teasing in a hurtful way and by threatening or forcing others to do things they did not want to do.Being called mean names, made fun of or teased in a hurtful way was the most reported specific form of victimization. Consistent with our results, verbal bullying and victimization were the most prevalent forms reported by students^21,22^. The expression of violence in its verbal form (e.g., name calling and threats) was most frequently present in the relationships between girls, while boys are involved more directly in the phenomenon using physical violence (e.g., hitting, pushing, and kicking) ^23^.

Compared to the other groups (pure victims, pure bullies, and bystander), the bully-victims were the most likely to report low empathy, fear of being bullied, tendency to aggressiveness, disliking school and having fewer friends.

Identifying emotional and behavioral problems among bullies, victims, and bully-victims is very challenging because the participant roles can be interchangeable ^24^. Generally, the victims experience more emotional problems including depression, loneliness, and fear of being victimized; the bullies experience more behavioral problems such as aggressiveness and low empathy; the bully-victims tend to experience both emotional and behavioral problems and share characteristics of both the bullies and the victims ^25^. The bully-victims generally experience the most problems and had the highest risk of adverse outcomes^24,25^. Bully-victims have generally fewer friends than other victims or even bullies. Bully-victims are even more peer rejected than pure victims and they lack the support of friendship known to have a protective effect on victimization. In addition to the internalizing and externalizing problems, students involved in bullying frequently reported disliking school. The bully-victims were represented the largest percentage of students who reported disliking school (13.6%) compared to the victims (10.0%) and to the non-bullied students (6.4%) ^26^. The school environment may be especially difficult for these students.

Based on our findings, most of the bullies reported that they did not talk about their bullying others with an adult. Only 30.3% of the victims have reported that they told someone of their victimization and most frequently, it was a friend.

The politic of “telling” is a critical component of anti-bullying policies and appropriate preventive strategies. However, students tell others about bullying relatively infrequently, with greater numbers choosing to share information with friends or parents comparing to teachers^27^. Consistent with our results, the likelihood of telling friends remained high compared to telling parents and teachers, suggesting that telling friends is perceived as a less risky option^27^. About 53% of youth were told their teachers and 67% told their parents^10^. Bullied pupils who tell someone about their being bullied were more likely to tell family members (45%) or friends (43%), than school staff (35%) ^28^. Children's reluctance to talk to adults about bullying may have numerous reasons including ineffective, insensitive or excessive perceived adult responses and the influence of peer cultures discouraging “telling” to adults ^27^. Students may attribute their reluctance to tell their teachers to a worry that their reports might be dismissed as inaccurate or unbelievable, a fear that teachers might reveal their report or a feel that telling a teacher will worsen the situation ^27,29^. Children are also reluctant to tell their parents about bullying, because they may feel ashamed and rejected, do not want to worry their parents or worry about an overreaction of their parents ^27^. In addition, instead of implicating adults in their bullying problems, most students choose other solutions such as ignoring, reciprocating or fighting ^29,30^. A substantial culture shift in attitudes children and adults toward the politic of 'telling' is hence needed. Until this happens, confidential helplines were an essential resource for children and young people for more effectiveness in reducing and preventing bullying^27^.

Many of the teachers and parents did not talk to the bullies about their behavior^10^. This could be because they did not know about the incidents or did not know what to do ^10^. Our results showed that, most frequently, the middle school students perceived that teachers and classmates did nothing to counteract the bullying behavior. The majority of the victims indicated that their parents never contacted the school to stop their victimization.

Teachers did not respond in 27% of the cases when they saw bullying occurring^31^. The lack of sensitivity to or awareness of ‘covert’ activity could explain the teachers’ limited response to bullying in classroom settings ^31^. The role of teacher is critical in prevention efforts of the bullying problem In a qualitative study assessing the perception of parents and teachers of peer bullying conducted in Iran, authors recommended that teachers should participate in antibullying programs and build supportive relationship with parents to prevent this problem ^32^.

In relation to parental response to bullying, most of parents who were aware of their children victimization did not intervene^33^. However, parents can face a number of difficulties that influence partnerships with the school to tackle the bullying problem^34^. Parents had reported problems in contacting teachers directly to report their complaint^35^.

In reference to peer response to bullying, 36.3% of bullied students reported that their classmates did nothing to stop bullying^10^. Although peers are often part of the bullying problem, they can also be part of the solution ^36^. Anti-bullying interventions should target peer bystanders’ by enhancing their empathy toward the victims and by raising their self-efficacy to defend them ^36^.

Bystanders are the most prevalent group compared to pure victims, pure bullies, and bully-victims in the current study. Bystanders are likely to be easier to influence than the active bullies^36^, one could expect a reduction in the prevalence of bullying in our middle schools by targeting bystanders in the prevention efforts.

Because parents, teachers, and classmates may influence the bullying problem, they should be included in prevention strategies. The most effective programs are ones that have a multi-level systemic approach^37^ taking into account the social-ecological model of bullying which postulates that bullying behavior is determined by the multiple systems in which youth belong^38^.

Our study has some limitations. We investigated the prevalence of bullying using self-report that led to missing data in some items across the questionnaire. This is a common problem in self-report studies^39^. Furthermore, our study was conducted in only one Tunisian governorate rather than multiple governorates. Despite these limitations, our study has strengths. It consists mainly of the large sample which increases the precision of our estimates and the power of the study to draw conclusions. This is also the first Tunisian study focusing on the phenomenon of bullying.

## Conclusions

To be able to intervene to prevent bullying, it is important to recognize the problems of the students facing the bullying behavior as victim or perpetrator. Most of students perceived that classmates and teachers did nothing to counteract bullying and talking about bullying was not a frequent pattern among them. Preventive efforts should begin by opening the line of communication between students, their parents, their teachers and their friends since talking is the first step to understand this behavior. Bullying is a systemic problem involving multiple contexts such as individual students, peers, teachers, and parents. Hence, through effective anti-bullying programs, they should learn effective ways to handle this problem.

## Ethical considerations

Permission to carry out the survey was obtained from the Ministries of Health and Education. Informed parental consent to participate in the study was collected before beginning the study. The school managers gave us permission to conduct the survey. Confidentiality was upheld by allowing for anonymity in completing the questionnaire.

## Acknowledgments

Authors thank the Ministries of Health and Education and the staff of schools where study was undertaken. We also thank participants and their parents.

## Conflict of interest statement

The authors declare that there is no conflict of interest.

## Funding

None.

## Highlights

- Boys were more likely to be pure bullies.
- Girls were more likely to be involved in bullying verbally .
- Talking to someone about bullying was not a frequent pattern among students .
- Most of the middle school students perceived that classmates and teachers did nothing to counteract bullying .
- The bully-victims were more likely to experience emotional and behavioral problems compared to other groups.
